# Kinetics of the Glass Transition of Silica-Filled Styrene–Butadiene Rubber: The Effect of Resins

**DOI:** 10.3390/polym14132626

**Published:** 2022-06-28

**Authors:** Niclas Lindemann, Jürgen E. K. Schawe, Jorge Lacayo-Pineda

**Affiliations:** 1 Institut für Physikalische Chemie und Elektrochemie, Leibniz Universität Hannover, Callinstraße 3A, 30167 Hanover, Germany; 2 Continental Reifen Deutschland GmbH, Jädekamp 30, 30419 Hanover, Germany; jorge.lacayo-pineda@conti.de; 3 Mettler-Toledo GmbH, Heuwinkelstrasse 3, 8606 Nänikon, Switzerland; juergen.schawe@mt.com; 4 Institut für Anorganische Chemie, Leibniz Universität Hannover, Callinstraße 9, 30167 Hanover, Germany

**Keywords:** glass transition, kinetics, rubber, resin, BDS, FDSC

## Abstract

Resins are important for enhancing both the processability and performance of rubber. Their efficient utilization requires knowledge about their influence on the dynamic glass transition and their miscibility behavior in the specific rubber compound. The resins investigated, poly-(α-methylstyrene) (AMS) and indene-coumarone (IC), differ in molecular rigidity but have a similar aromaticity degree and glass transition temperature. Transmission electron microscopy (TEM) investigations show an accumulation of IC around the silanized silica in styrene–butadiene rubber (SBR) at high contents, while AMS does not show this effect. This higher affinity between IC and the silica surface leads to an increased compactness of the filler network, as determined by dynamic mechanical analysis (DMA). The influence of the resin content on the glass transition of the rubber compounds is evaluated in the sense of the Gordon–Taylor equation and suggests a rigid amorphous fraction for the accumulated IC. Broadband dielectric spectroscopy (BDS) and fast differential scanning calorimetry (FDSC) are applied for the characterization of the dielectric and thermal relaxations as well as for the corresponding vitrification kinetics. The cooling rate dependence of the vitrification process is combined with the thermal and dielectric relaxation time by one single Vogel–Fulcher–Tammann–Hesse equation, showing an increased fragility of the rubber containing AMS.

## 1. Introduction

The properties of elastomer-based materials can be modified by blending different polymers [1,2,3] and mixing them with various additives, such as fillers [4,5], plasticizers [6,7,8] and different vulcanization systems [9,10,11,12] for a wide variety of technical applications. A frequently used form of modification is the coupling of the rubber matrix with reinforcing fillers in order to tailor the mechanical properties to the application [4]. Apart from carbon black as a conventional filler, precipitated silica with a silane coupling agent is state-of-the-art in tire compounds [4,13]. The advantage of silica arises with an adaption of the polymer to solution styrene–butadiene rubber (SBR) [14,15]. The silica-filled rubber provides a lower rolling resistance and higher wet traction without decreasing the abrasion resistance [13].

High amounts of fillers can disturb the processability of rubber compounds due to their higher viscosity. Oils and resins are used to counteract this rheological behavior. Additionally, the tackiness of the rubber compounds can be increased by some types of resins [16,17,18]. Hydrocarbon resins, with a high glass transition temperature, *T*_g_, and a melting point, *T*_m_, at the processing temperature are beneficial in preserving the rubber compound hardness at the service temperature [16]. This is where the possibility to decrease the rolling resistance of a tire or to lower fuel consumption occurs and therefore, contributes to a reduction in CO_2_ emissions. On the other hand, the hardness of the rubber compound does not necessarily decrease the braking performance to the same degree. The rolling resistance mainly correlates with a dynamic excitation at low frequencies of around 100 Hz, while higher frequencies of around 10^5^ Hz are characteristic of traction [19]. Hence, the material properties at different frequencies are important parameters, which are strongly linked to molecular dynamics and the local structure in the elastomer system. The glass transition is a phenomenon sensitive to molecular dynamics. Its modification, due to local structural changes, is, therefore, the focus of many investigations [19,20]. Two manifestations are characteristic of the glass’ transition: (i) the relaxation process, measured by frequency-dependent dynamical experiments in the rubbery state, which is also called “dynamic glass transition”; and (ii) the vitrification process, occurring during cooling as the transformation from a soft rubbery state into a solid glassy state [19].

The addition of plasticizers in the rubber matrix increases the flexibility of the polymer chains and usually decreases the *T*_g_ of the rubber compound [8,21]. The influence on the dynamic properties depends on the specific combination of plasticizer and rubber. For a flexible plasticizer having a small molecular size, the strength of the attractive interactions between the polymer and the plasticizer is of great importance for the dynamical glass transition [22]. In contrast to plasticizers, resins usually increase the *T*_g_ of the rubber compound [23,24]. Furthermore, the miscibility between resin and the host polymer is more often crucial [25].

With the addition of nanosized filler particles, the rubber compound becomes a polymeric nanocomposite showing additional interfacial phenomena. The surface of silica fillers mostly leads to a reduced mobility with a slower relaxation process of the host polymer [26,27,28,29]. The enhanced properties of the rubber compound are related to these interfacial interactions [30,31]. The interfacial effects result from both the interactions between the host polymer and the silica fillers (polymer–filler interaction) and interactions between the silica fillers among each other (filler–filler interaction). To increase the compatibility between silica and the host polymer, surface modifications of the silica are necessary [4,32]. Increasingly, the host polymer is functionalized as well [33,34]. Filler–filler interactions are necessary to build a network structure which provides reinforcing properties. Besides the surface modification, the surface area of the particles is critical for the mechanical properties of the rubber compound [35,36].

In this study, we characterize the variations in the molecular dynamics of a silica-filled styrene–butadiene rubber (SBR) system, which is mixed with two different resins: poly-(α-methylstyrene) (AMS) and indene-coumarone (IC). These resins differ in rigidity [37] but have a similar aromaticity degree and glass transition temperature (*T*_g_ ≈ 45 °C). The efficient use of the resins depends on the miscibility between the resin and the polymer.

The morphology of the resulting rubbers is investigated by transmission electron microscopy (TEM). The influence of the composition on the relaxation behavior and glass transition is evaluated by conventional differential scanning calorimetry (DSC), fast differential scanning calorimetry (FDSC), temperature-modulated FDSC and broadband dielectric spectroscopy (BDS).

Dynamic glass transition takes place in the structurally equilibrated super-cooled melt as a thermal relaxation process, characterized by the relaxation time, *τ*, and the dynamic glass transition temperature *T*_g,ω_ [38]. During vitrification, the structurally equilibrated super-cooled melt transforms into a non-equilibrated glassy state. This transformation depends on the cooling rate *β*_c_ [19,39] and correlates with the relaxation time [40]. The correlation between *β*_c_ and *τ* has been described for thermoplastics [41,42,43] and unfilled solution styrene–butadiene rubber (SBR) [44] elsewhere, and is valid for the silica-filled SBR used in this study.

In this article, we investigate the influence of AMS and IC on the glass transition and the kinetics of relaxation and vitrification in vulcanized-SBR filled with silica. Furthermore, the affinity of the resin to accumulate at the silanized silica surface and the consequences for the filler network are studied.

## 2. Materials and Methods

### 2.1. Materials

**Figure 1:**
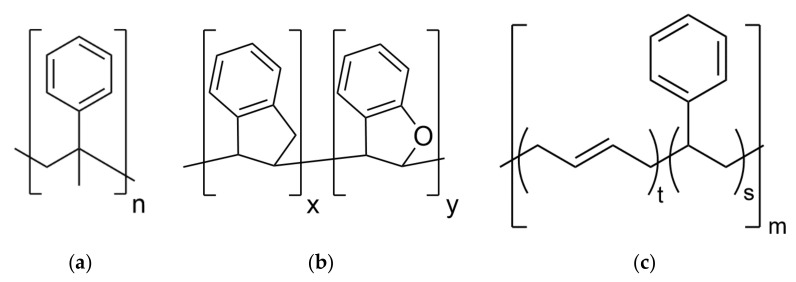
(**a**) Chemical structure of poly-(α-methylstyrene) (AMS), *n* ≈ 10; (**b**) chemical structure of indene-coumarone (IC) resin x + y ≈ 10 with a proportion of 95% indene and 5% coumarone; (**c**) chemical structure of styrene–butadiene rubber (SBR). Styrene groups (s), chain part in trans-orientation (t), m > 6000.

The materials for this investigation are the solution styrene–butadiene rubbers (SBR) vulcanized with sulfur and filled with silica. They consist of a systematic variation in resin content. The resins are poly-(α-methylstyrene) (AMS) and indene-coumarone (IC). The chemical structures of SBR, AMS and IC are shown in Figure 1.

The formulations are given in Table 1. It is common practice in the rubber industry to develop compound formulations using the non-SI unit “parts per hundred rubber” (phr) for the weight of a component per 100 units of rubber. The relation between phr and the weight percentage for a component i is given by

(1) $$\mathrm{wt}{\%}_{i}={\mathrm{phr}}_{i}/\sum_{j}{\mathrm{phr}}_{j}$$

and shown for the resins in Table 2.

### 2.2. Mixing and Vulcanization

The ingredients were mixed in a two-step mixing process with a 300 mL miniature internal mixer Haake Rheomix (Thermo Fisher Scientific, Waltham, MA, USA). In the first step, all ingredients, except the vulcanization system (DPG, CBS and sulfur), were mixed at around 140 °C for 3 min. After adding the vulcanization system in the second step, the rubber compound was mixed at 80 °C for 3 min to avoid premature crosslinking. Afterwards, the samples were vulcanized at 160 °C, according to *t*_90_, the time for the 90% crosslinking, as listed in Table 3. The *t*_90_ time was determined according to ASTM D5289 [45].

### 2.3. Methods

#### 2.3.1. Broadband Dielectric Spectroscopy (BDS)

The dielectric measurements were performed with an Alpha-A High-Performance Frequency Analyzer with a Novocool cryo-system (Novocontrol Technologies, Montabaur, Germany). The isothermal frequency sweeps, between 0.1 Hz and 2 × 10^6^ Hz, were performed in a temperature range from −100 °C to 70 °C with an increment of 5 K. Specimens with a thickness from 150 µm to 250 µm were mounted between two round gold-plated electrodes in a plate-capacitor arrangement with a diameter of 30 mm.

#### 2.3.2. Conventional Differential Scanning Calorimetry (DSC)

Conventional DSC measurements were performed with a DSC 1 (Mettler-Toledo, Greifensee, Switzerland) equipped with the liquid nitrogen cooling option and the HSS-8 sensor. The device was adjusted with n-octan, water, indium and zinc. The scanning rate was 10 K/min in a temperature range between −140 °C and 40 °C. The specimen was cooled and subsequently heated. In between these scanning segments, the instrument was equilibrated for 3 min. The specimens were prepared as cylindric sheets with a thickness of about 0.3 mm and a diameter of 4 mm. They were measured in a hermetically sealed standard Al-crucible.

#### 2.3.3. Fast Differential Scanning Calorimetry (FDSC)

The FDSC experiments were performed using a Flash DSC 1 (Mettler-Toledo, Greifensee, Switzerland) equipped with an Intracooler TC100 (Huber, Offenburg, Germany) to reach the low temperature needed for the analysis of the glass transition in elastomers. The UFS 1 sensor was purged with a 20 mL/min nitrogen gas. The sensor’s support temperature during the measurement was set at −95 °C.

Samples of the rubber compounds with a resin content of up to 40 phr were prepared as slices of 6 µm thickness using a cryo-microtome MT-990 (RMC Boeckeler, Tucson, AZ, USA) equipped with a glass knife operated at −60 °C and a cutting speed of 1 mm/s. The microtomic slices were cut with a scalpel to attain a final specimen shape smaller than (150 μm)^2^, which is comparable to the area of the center of the active zone of the sensor. The stickier specimens, prepared from the rubber compounds with higher resin contents, were first shaped in the cryo-microtome using an angulated diamond knife. A slice of 6 µm thickness was cut and carefully placed on the chip sensor, which was stored inside the cryo-chamber of the microtome. In this way, flat and thin specimens were produced that exhibited a good thermal contact when placed within the active zone of the chip sensor [46].

The prepared specimens were cooled from 40 °C to −95 °C at rates between 1500 K/s and 0.1 K/s, and were subsequently heated at a rate of 1000 K/s to determine the cooling rate dependence of the glass transition. The glass transition temperature is defined as the limiting fictive temperature [47,48,49]. To evaluate the thermal contact between the specimen and sensor, measurements with a cooling and heating rate of 1000 K/s were performed for each specimen. As expected for a sufficient thermal contact, the fictive temperatures that were measured during the cooling and subsequent heating were identical within the limits of experimental uncertainty. Thus, the preparation was considered to be successful [47].

**Figure 2:**
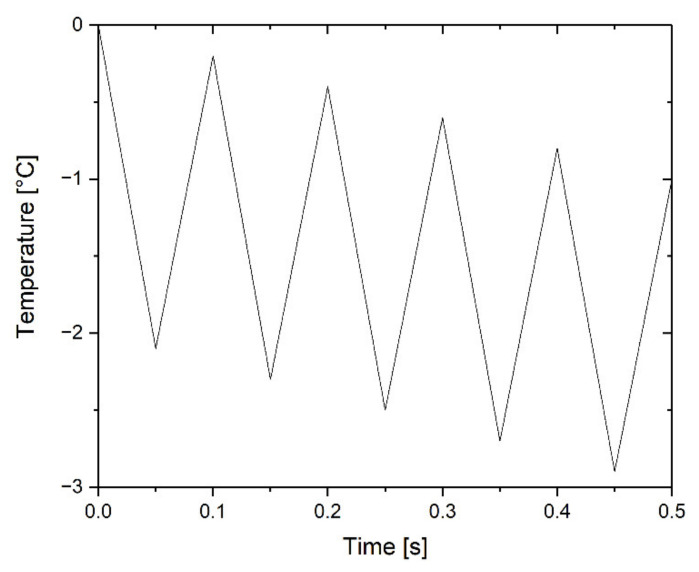
Sequence of the temperature program for temperature-modulated fast differential scanning calorimetry (TM-FDSC).

Temperature-modulated fast differential scanning calorimetry (TM-FDSC) was performed for the selected specimens using a sawtooth-modulation function (Figure 2). The temperature amplitude was 2 K, and the period was 0.1 s. The underlying cooling rate was −2 K/s between 0 °C and −60 °C. The TM-FDSC measurements were evaluated using the first harmonic of Fourier analysis.

The resulting scanning rates were fast enough to obtain a suitable signal and slow enough to achieve a high resolution without any smearing effects (see ref. [41]). The temperature program was devised as a sequence of heating and cooling steps and were calculated as follows: (1) Cooling step of 2.1 K with a cooling rate of −42 K/s; (2) heating step of 1.9 K with a heating rate of 38 K/s; (3) repetition of steps 1 and 2 until the lowest temperature of −60 °C is reached.

The calibration of the sensor was performed with a post-measurement calibration using adamantane as a reference substance. Further details on the sample preparation and calibration are given in ref. [44].

#### 2.3.4. Transmission Electron Microscope (TEM)

The TEM investigation was performed on a JEM-1400 (Jeol, Tokyo, Japan) using an acceleration voltage of 100 kV. Specimens of 60 nm thickness were cut with a cryo-ultramicrotome Leica EM UC6/EM FC6 (Leica Microsystems, Wetzlar, Germany) equipped with a diamond knife. The cutting temperature was −55 °C.

#### 2.3.5. Dynamic Mechanical Analysis (DMA)

DMA investigations of the vulcanized specimens were performed in compression mode on a DMA Gabo Eplexor^®^ 150N (Netzsch, Ahlden, Germany). Strain sweeps between 0.1% and 12% and at a frequency of 10 Hz were performed at 55 °C with a static strain of 20%. The samples were prepared as cylindrical specimens with a diameter and height of 10 mm, respectively. 

## 3. Results and Discussion

### 3.1. Structural Investigation

**Figure 3:**
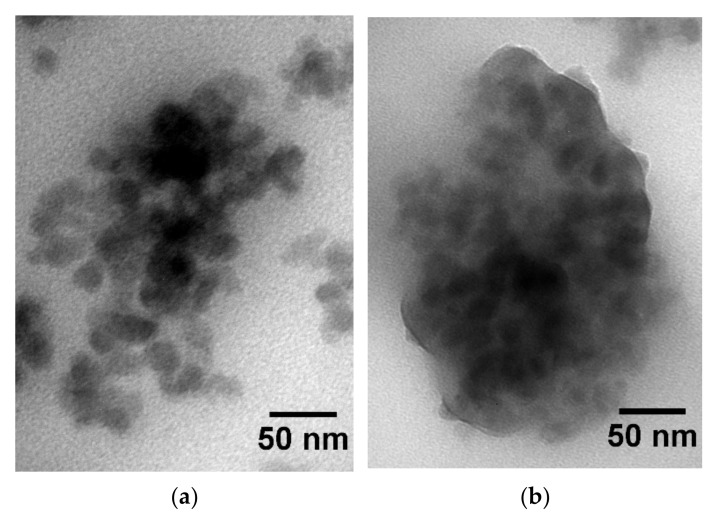
(**a**) TEM image of the rubber compound containing 80 phr AMS as resin; (**b**) TEM image of the rubber compound containing 80 phr IC as resin.

The structure of the rubber compounds at high concentrations of resin was visualized using TEM imaging. Figure 3a,b show the TEM images of the rubber compounds containing 80 phr AMS and IC, respectively. The image of the rubber compound containing AMS (Figure 3a) shows a homogenous matrix with silica-filler particles forming aggregates in the matrix. The AMS is indistinguishable from the polymer. In the case of the IC compound (Figure 3b), the silica-filler particles are surrounded by a substance of 5 to 10 nm thickness. 

**Figure 4:**
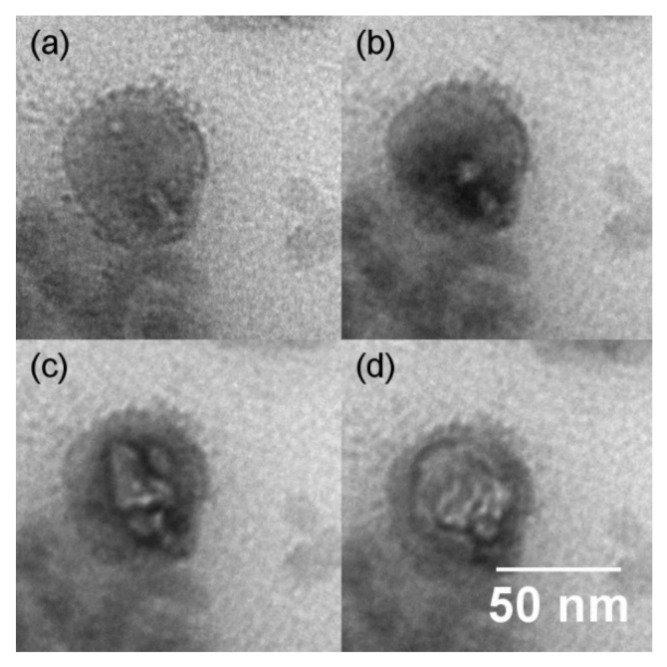
TEM image of the rubber compound containing 80 phr IC as resin showing the organic matter being sensitive to beam damages. The images were taken after different times of radiation treatment. (**a**) shows the untreated sample, and between (**b**–**d**), the treatment time was extended by 5 s each.

To identify this substance, the filler particles were irradiated with the focused electron beam of the TEM. The substance around the filler particles was easily damaged (Figure 4), as is known for organic matter. While the primary damage mechanism is caused by inelastic scattering, the damage of the organic substance is due to heat and bond scission [50]. This organic substance in the rubber compound containing IC tends to accumulate at the silica–polymer interface. It has an affinity for the silica particles.

### 3.2. Linearity of the Mechanical Response

**Figure 5:**
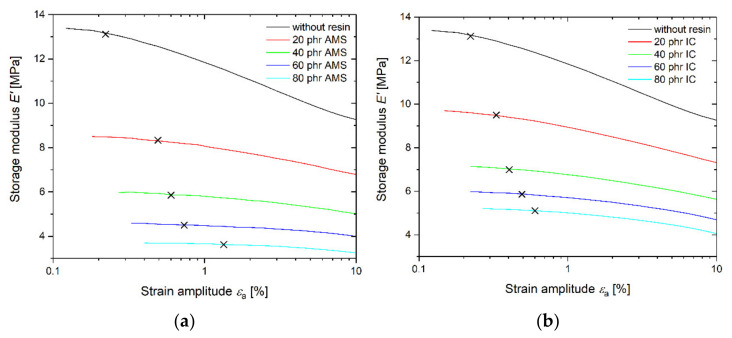
(**a**) Strain sweeps of AMS compounds; (**b**) strain sweeps of IC compounds. The linearity limit is indicated on the curves.

**Figure 6:**
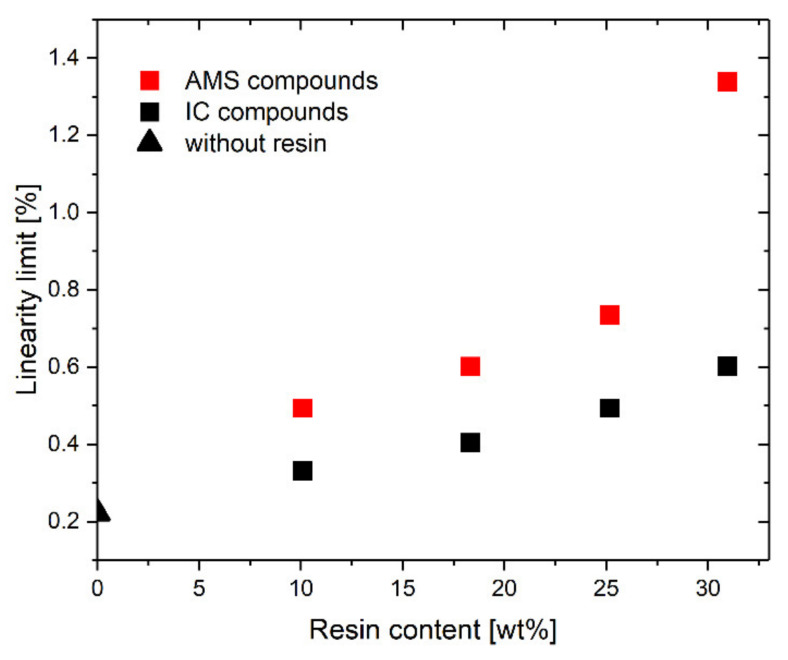
Linearity limit of the mechanical response as a function of the resin content.

Rheological linearity occurs when the modulus is invariant with respect to the strain amplitude. Elastomers containing reinforcing fillers show a decrease in the dynamic storage modulus, *E*′, with an increasing strain amplitude, *ε*_a_ (Payne-effect) [4,51,52]. The *E*′*-ε*_a_ diagram for both the AMS (a) and the IC rubber compounds (b) is displayed in Figure 5. As expected, the modulus decreases with the increasing resin content. The linearity limit, indicated on the curves in Figure 5, is defined as the strain amplitude at which *E*′ is reduced by 2%. This limit is always lower for SBR-IC (Figure 6).

The nonlinear behavior is due to the disruption of the filler–filler network and, therefore, is related to the percolation threshold [53]. Syed et al. showed a reduced filler percolation threshold for carbon black filled rubber with an increasing resin content [54]. The resin interacts with the surface of the filler, acts as an activator, and builds a more compact filler network [54].

For the rubber compound in this study, the IC that accumulated at the silica surface likely acts in a similar way and led to a more compact filler network. This higher compactness of the filler can lead to a stronger nonlinearity of the SBR-IC, as shown in Figure 5 and Figure 6.

### 3.3. Composition Dependence of the Glass Transition 

**Figure 7:**
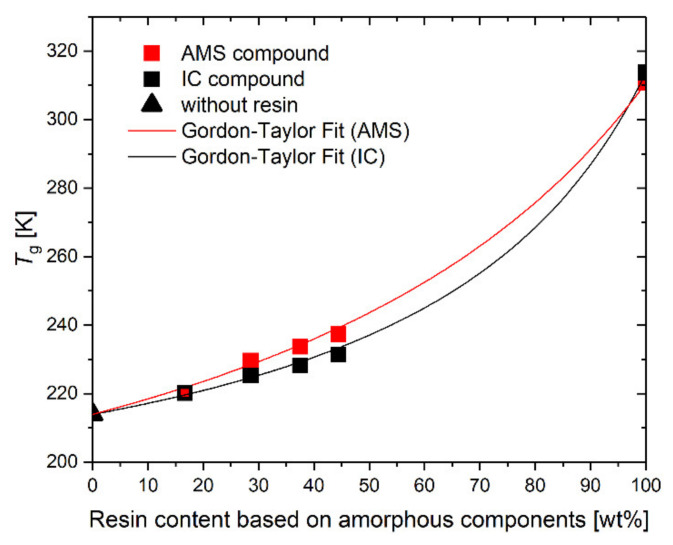
Glass transition temperatures of the different rubber compounds as a function of the resin content in relation to the amorphous components. The lines represent the Gordon–Taylor fits.

The glass transition temperatures, *T*_g_, are measured by DSC at a cooling rate of 10 K/min. As shown in Figure 7, *T*_g_ increases with the increasing resin content. For the determination of the weight fraction, only the amorphous components (polymer and resin) are considered. The initial slope in the diagram in Figure 7 is larger for the SBR-AMS compared with the SBR-IC. Similar behavior was found for the AMS and IC in polybutadiene rubber [37,55]. The glass transition dependence of a mixture of amorphous components is usually described by the Gordon–Taylor (GT) equation [56,57]:

(2) $$T_{g,\mathrm{mix}}=\frac{w_{c}T_{g,c}+kw_{r}T_{g,r}}{w_{c}+kw_{r}}$$

where *w* stands for the weight fractions and *T*_g_ for the glass transition temperatures, the indices c and r refer to the polymer components and the pure resin, respectively. The GT parameter *k* is a fitting parameter. The fitting curves using Equation (2) are shown in Figure 7. The values of the GT-parameters are calculated as *k*_fit,IC_ = 0.30 for the SBR- IC and *k*_fit,AMS_ = 0.44 for the SBR-AMS.

For the athermic mixtures, the GT parameter is [58]:

(3) $$k=\frac{\Delta c_{p,r}}{\Delta c_{p,c}}.$$

With the intensity of the glass transition for SBR, $\Delta c_{p,c}=0.51\;$J/gK, the calculated *k*_calc_ values are obtained and listed in Table 4.

Both resins show significant differences between *k*_fit_ and *k*_0_. Hence, the specific molecular interactions between the resin and the polymer are expected [59], resulting in the rubber compounds being thermic mixtures. The difference between *k*_fit_ and *k*_0_ increases for the SBR-IC compared to the SBR-AMS. This could be a consequence of the stronger molecular interactions between the SBR and IC, or a decreased effective resin content in the polymer-resin mixture caused by the increased amount of IC at the silanized silica surface (Figure 3 and Figure 4). However, the reduced IC content is most likely not sufficient for the large difference in *k*_fit_.

**Figure 8:**
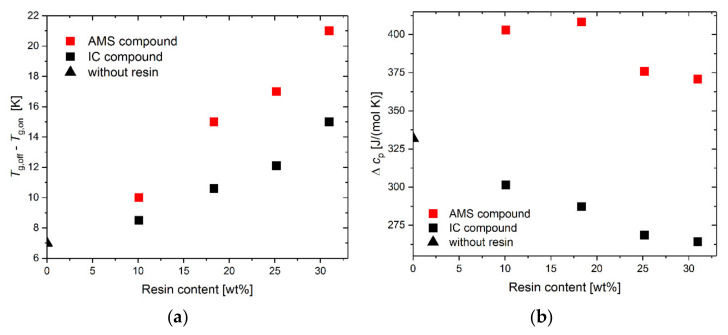
(**a**) Width of the glass transition determined as the difference between the onset and the offset as a function of the resin content. (**b**) Intensity of the glass transition as a function of the resin content.

The increase in the width of the calorimetric glass transition, Δ*T*_w_, with an increasing resin content (Figure 8) is stronger for the SBR-AMS compared with SBR-IC. Besides the effect of the reduced effective IC content, the IC is expected to have stronger specific molecular interactions with the SBR compared to AMS. The width of the calorimetric glass transition can be understood as a more reliable value for the determination of the miscibility behavior in the polymer blends compared to the shift in the glass transition temperature [60].

The width of the calorimetric glass transition is related to the average temperature fluctuation in the cooperative rearrangement regions (CRR) [40]. The size of those regions decreases with an increasing temperature fluctuation, and consequently, the size of the CRR is expected to be bigger for the IC compound compared to the AMS compound at the same resin level [61]. The interactions of IC with the polymer might yield a decrease in the volume of the independently movable regions, the CRRs. This effect is less pronounced for AMS. Thus, the less flexible IC in the SBR matrix may reduce the mobility of the polymer chain segments responsible for the glass transition more than AMS at the same content. Since the aromaticity degree and the glass transition temperature of both resins, AMS and IC, are very similar, it can be assumed that the reduced interactions are due to the differences in their molecular rigidity.

The intensity of the glass transition, Δ*c_p_*, decreases in the case of the SBR-IC, while AMS increases the intensity of the glass transition (Figure 8b). The decrease in Δ*c_p_* of the SBR-IC indicates a reduced contribution of amorphous material for this glass transition. In the case of partial-phase separation, a second glass transition at higher temperatures, or at least a significant broadening of the glass transition, is expected. Such behavior was not found. The accumulation of the IC-based material, together with the decrease in Δ*c_p_*, indicates the formation of a rigid amorphous fraction on the silica surface [27,62].

### 3.4. Dielectric Relaxation

**Figure 9:**
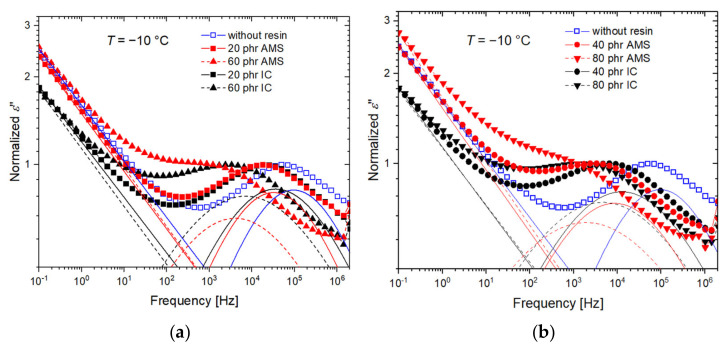
Dielectric losses as a function of the frequency normalized to the peak maximum of the α-relaxation for all rubber compounds measured at −10 °C. (**a**) and (**b**) show curves of samples with different resin contents in two groups to improve visibility. The fits, according to Equation (6) of the conductivity contributions and the relaxation processes, are indicated separately.

To characterize the relaxation behavior in a wide frequency range, dielectric measurements were performed. The dielectric loss *ε*” is normalized to the peak maximum and plotted in Figure 9 as a function of the angular frequency *ω* at −10 °C for all rubber compounds under investigation. The peak is caused by the α-relaxation. The peak frequency decreases with the increasing resin content. The peak shift is stronger for the SBR-AMS compared with SBR-IC. 

The decay of the curves at low frequencies is caused by both the contribution of conductivity

(4) $$\sigma \left(\omega \right)=\frac{\sigma_{0}}{i\omega \varepsilon_{0}}$$

and the Maxwell–Wagner–Sillars relaxations, which are triggered by the tapping of the charge carriers at the silica/polymer interface [26,63,64,65,66,67]. The latter effect can be taken into account in the dielectric loss equation by adding the exponent *N* to the frequency dependence of the conductivity contribution resulting in [28,68]

(5) $$\sigma \left(\omega \right)={\left(\frac{\sigma_{0}}{i\omega \varepsilon_{0}}\right)}^{N}.$$

The accumulation of charge carriers at the interface can lead to a formation of a high dipole moment [65,69]. This leads to strong signals in the BDS measurement compared to the rubber compounds having a low polarity.

Symmetric relaxation processes, such as the α-relaxation in SBR [10,70], can be described by the Cole–Cole equation with a shape constant α. The complex permittivity function can be described by

(6) $$\varepsilon^{*}(\omega )=\varepsilon_{\infty }+\frac{\Delta \varepsilon }{1+{\left(i\omega \tau \right)}^{\alpha }}+{\left(\frac{\sigma_{0}}{i\omega \varepsilon_{0}}\right)}^{N}$$

where i is the imaginary unit, *ε*_∞_ is the high-frequency limit of the permittivity, ∆*ε* is the relaxation strength, and *τ* is the characteristic relaxation time. The characteristic relaxation time *τ* can be determined from the peak maximum of the dielectric loss peak by *ω*_max_*τ* ≈ 1, where *ω*_max_ is the angular frequency at the maximum of the fitted relaxation function.

The vulcanization accelerator, DPG, is known to show a dielectric response that is slightly slower compared to the α-relaxation of SBR, which is possibly coupled to the segmental dynamics of the polymer [10,71]. For the silica-filled rubber compounds, DPG is assumed to be adsorbed by silica, which decreases the relaxation strength of this slow process [10]. Together, with the increasing strength of MWS and conductivity contribution, the slow process becomes indistinguishable within the curves.

### 3.5. Thermal Relaxation

#### 3.5.1. Temperature Modulation

Thermal relaxation was measured by temperature-modulated DSC (TM-DSC) using the approach of the frequency-dependent complex heat capacity [72,73]

(7) $$c_{p}{}^{*}\left(\omega ,T\right)=c_{p}^{\prime }\left(\omega ,T\right)-i\;c_{p}^{\prime \prime }\left(\omega ,T\right).$$

**Figure 10:**
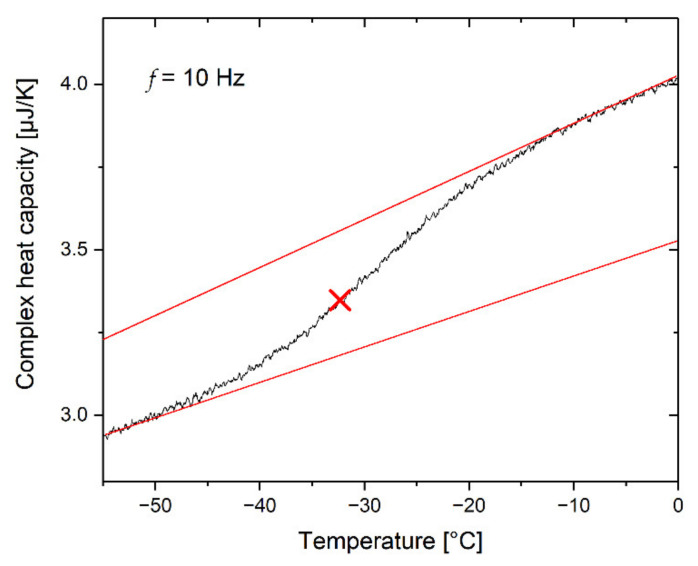
Complex heat capacity curve of the rubber compound containing 80 phr AMS. The intersection point at *T*_g_ = −32.3 °C is indicated.

The FDSC measurements were performed by means of sawtooth modulation. The evaluation was carried out by Fourier analysis of the first harmonic at a frequency of *f* = 10 Hz and an underlying cooling rate of 2 K/s. As an example, the complex heat capacity component c_p_* of the rubber compound containing 80 phr AMS is shown in Figure 10. The characteristic relaxation time is *τ* = 1/(2 π *f*) = 16 ms. The respective temperature is taken from the inflection point of the c_p_*(*T*) curve.

#### 3.5.2. Vitrification

The cooling rate dependence of the glass transition characterizes the thermal relaxation behavior [40]. The characteristic glass temperature, *T*_g_, of the vitrification is indicated by the limiting fictive temperature, *T*_f_:

(8) $$T_{g}=T_{\mathrm{rl}}-\overset{T_{\mathrm{rl}}}{\underset{T_{\mathrm{rg}}}{\int }}\frac{\phi \left(T\right)-\phi_{g}\left(T\right)}{\phi_{l}\left(T\right)-\phi_{g}\left(T\right)}dT,$$

where *ϕ*(*T*) is the measured heat flow curve, *ϕ*_l_(*T*) is the extrapolation of the liquid state, and *ϕ*_g_(*T*) is the extrapolation of the glassy state. *T*_rl_ and *T*_rg_ are the reference temperatures in the super-cooled liquid and glassy state, respectively [74,75].

The cooling rate dependence of *T*_g_ is measured in a range between 0.1 and 1500 K/s. To determine *T*_g_, the specimens were subsequently heated at 1000 K/s. This method can be applied because the limiting fictive temperature of the heating curve is identical to that of the previous cooling if no aging in the glassy state occurs. This is a consequence of the conservation of energy [76].

**Figure 11:**
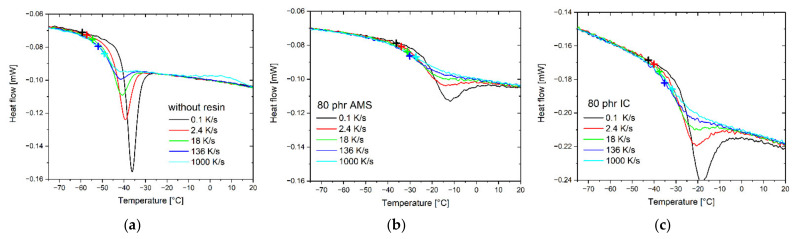
Selected FDSC curves at 1000 K/s measured after cooling at the indicated rates (**a**) for the rubber compound without resins; (**b**) for the rubber compound containing 80 phr AMS; (**c**) for the rubber compound containing 80 phr IC. The glass transition temperatures are indicated.

Figure 11a shows the selected heating curves that were measured after cooling at different rates. As expected, *T*_g_ increases with an increasing cooling rate. Due to the hysteresis of the glass process, an overheating peak appears at the high-temperature side of the glass transition interval if the cooling rate *β*_c_ is lower than the heating rate *β*_h_ (|*β*_c_| < *β*_h_). The intensity of this peak increases with growing differences between the cooling and heating rates. The glass transition temperature, defined as the limiting fictive temperature, is a measure of the configurational entropy of the glass. Both properties decrease with the decreasing cooling rate.

Figure 11b,c show the selected heating curves that were measured after cooling at different rates for the rubber compounds containing 80 phr AMS and IC, respectively. In agreement with the conventional DSC measurements, both the shift and the broadening of the glass transition step increase stronger in the rubber with AMS compared with the IC. The enthalpic overshoot appears to be less pronounced for the rubber compounds containing 80 phr AMS compared with the sample containing 80 phr IC. This indicates a variation in the relaxation spectrum in the composites.

The cooling rate dependence of the glass transition temperatures shows differences between the two resins. The sample containing 80 phr IC exhibits a shift between the *T*_g_ measured after cooling at 1000 K/s and 0.1 K/s of 10.9 K. This is significantly larger than the same shift of the composite containing 80 phr AMS of 8.8 K.

### 3.6. Influence of the Composition on the Relaxation Kinetics

In the structurally equilibrated super-cooled liquid, the temperature dependence of the relaxation frequency 1/*τ* follows the Vogel–Fulcher–Tammann–Hesse (VFTH) equation [77,78,79,80]:

(9) $$\log\left(\tau^{-1}\cdot 1\;s\right)=A-\frac{B}{T-T_{V}}$$

where *A* is the logarithm of the pre-exponent factor, *B* is the curvature parameter and *T*_V_ is the Vogel temperature. The curvature parameter is related to the dynamic fragility *m* as [40,81,82,83,84]:

(10) $$m=\frac{BT}{{\left(T-T_{V}\right)}^{2}},$$

which describes the deviation from Arrhenius behavior.

**Figure 12:**
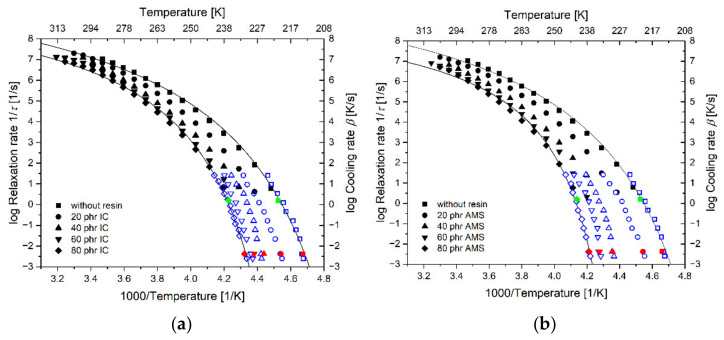
Activation diagrams of the different rubber compounds. The left ordinate is the logarithm of the reciprocal dielectric relaxation time of both BDS and TM-FDSC. The right ordinate is the logarithm of the cooling rate for both DSC and FDSC. The abscissa characterizes the measurement temperature of the dielectric measurements and the fictive temperature determined by the DSC and FDSC measurements, respectively. Data determined by: BDS (black) FDSC (blue), TM-FDSC (green), DSC (red). (**a**) Compounds containing IC; (**b**) compounds containing AMS. VFTH-fits are shown for the samples without resin and the samples containing 80 phr IC and AMS, respectively.

The activation diagram of the dielectric relaxation process is plotted in Figure 12. The DC conductivity and the Maxwell–Wagner–Sillars effect limit the measurement at low frequencies. The frequency range is, therefore, expanded using the data of the thermal relaxation. It has been shown for many materials that the activation curves of the dielectric permeability and the frequency-dependent dynamic heat capacity c_p_* are comparable [42,73,85,86].

The cooling rate dependence of the vitrification process is related to the thermal relaxation time [40,42,44,87,88]. For the thermo-rheologically simple materials, the relation between the relaxation time, *τ*, and the cooling rate of the vitrification process follows the Frenkel–Kobeko–Reiner (FKR) equation [40,44]:

(11) $$\log\left(\beta_{c}\tau /1\;K\right)=C$$

The logarithmic shift of *C* = 1.6 is determined by the best overlap between the cooling rate-dependent vitrification data and the dielectric and thermal relaxation frequencies, respectively. This fact agrees with our previous findings for the unfilled SBR without resin [44] and indicates the thermo-rheological simplicity of the investigated materials. Hence, the confinement effects do not play a role in the systems in this investigation [89]. 

The combined dataset describes the temperature dependence of the relaxation time in a wide range of about ten orders of magnitude and can be described by a single VFTH equation (Equation (9)). The fit parameters are listed in Table 5. Additionally, the fragility parameter *m* is determined at *T* = *T*_g_ using Equation (10).

**Figure 13:**
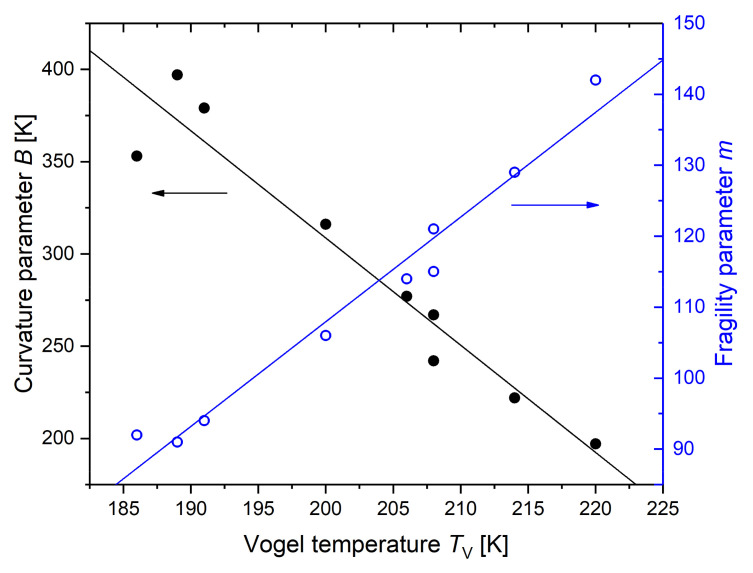
Indication of the linear correlation of both the curvature parameter and the fragility parameter with the Vogel temperature for the system of investigation.

The high-frequency limit for all rubber compounds is approximately identical (Figure 12), while the low-temperature limit (the Vogel temperature) differs with the changes in the composition. This leads to the assumption that the information of the variation in the Vogel temperature, *T*_V_, in the system of investigation, is comparable with that of curvature parameter *B* and the dynamic fragility *m*. The linear correlation between these parameters is shown in Figure 13.

**Figure 14:**
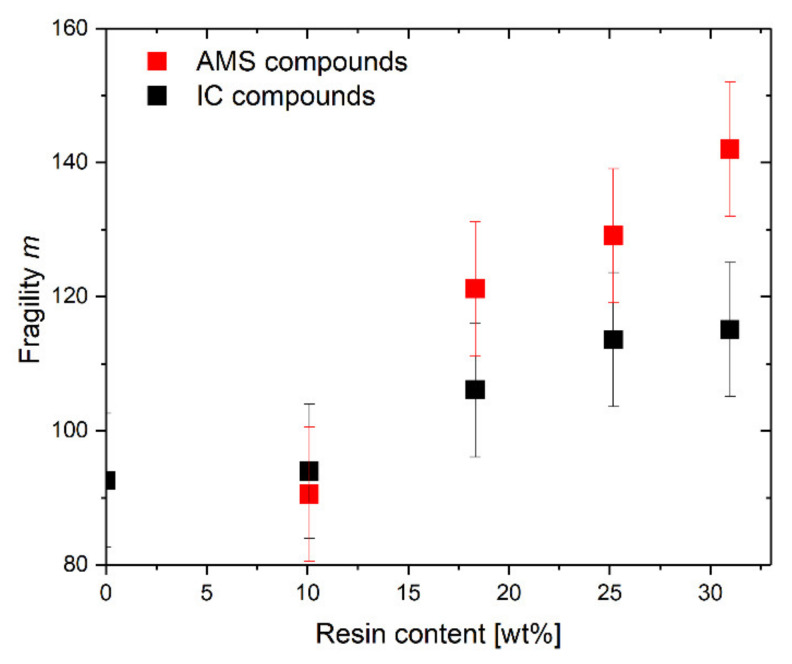
Fragility as a function of the resin content.

The fragility index *m* is plotted versus the amount of resin in Figure 14. A higher amount of resin leads to a higher dynamic fragility of the rubber compound. This effect appears to be less pronounced for the SBR-IC compared to the SBR-AMS and vanishes at high IC concentrations.

## 4. Conclusions

Resins are important additives in rubber compounds for enhancing both processability and the material properties. For efficient use, knowledge is needed about the effect of resins on the dynamic glass transition and the miscibility behavior in the rubber compound.

The resins AMS and IC, having a similar aromaticity degree and different molecular rigidity, are used as additives in vulcanized, silica-filled SBR. The structural investigations by TEM show an accumulation of IC around the filler particles at high contents, whereas no additional substance could be detected around the filler particles for the rubber compounds with AMS. The accumulation of IC on the silica particles generates a more compact filler network, which leads to a reduced filler percolation threshold, determined by the DMA measurements of the Payne-effect in a compression mode.

The phase diagram of the SBR-resin mixtures results in an increased difference between the theoretical GT parameter of an athermal mixture and the corresponding fit value. This indicates an increased specific interaction between the SBR and IC and, consequently, a higher affinity of IC to accumulate at the silica surface. The reduced intensity of the glass transition indicates the formation of an IC-enriched rigid amorphous fraction on the surface of the filler particles.

For both systems, the dielectric and thermal relaxation measurements result in the same activation curves, which differ depending on the type of resin and its content. The kinetics of vitrification were studied by the measurement of the cooling rate dependence of the glass transition by FDSC. According to the FKR equation, all activation curves of relaxation and vitrification overlap after shifting by the constant factor *C* = 1.6. This value agrees with the findings for unfilled SBR [44]. The validity of the FKR equation indicates thermo-rheological simplicity and enables the description of the glass process by a single VFTH equation in a frequency range of over ten orders of magnitude.

The effect of resin on the frequency dependence of *T*_g_ is strong at low frequencies, while the high-frequency limit is almost unaffected by the composition. This finding might open possibilities of efficiently tuning the material properties of rubber regarding the frequency response characteristics.

## Figures and Tables

**Table 1:** Formulation of the rubber compounds used in this study.

Ingredients	Quantity [phr ^1^]
SBR ^2^	100
Silica	60
TESPD ^3^	4.3
6PPD ^4^	2.0
Wax ^5^	2.0
Zink oxide	2.5
Stearic acid	2.5
DPG ^6^	1.0
CBS ^7^	2.0
Sulfur	2.0
AMS ^8^ or IC ^9^	0/20/40/60/80

^1^ Non-SI unit, parts per hundred rubber (phr); ^2^ microstructure: 30% cis, 28–32% vinyl, 15% styrene, 42% trans; ^3^ bis-[3-(Triethoxysilyl)-propyl]-disulfid; ^4^ N-(1,3-Dimethylbutyl)-N’-phenyl-p-phenylenediamine; ^5^ mixture of refined hydrocarbons and plastics; ^6^ 1,3-Diphenylguanidine; ^7^ N-Cyclohexylbenzothiazol-2-sulfenamid; ^8^ poly-(α-methylstyrene), *M*_w_ = 1296 g/mol, PDI = 1.78; ^9^ indene-coumarone (IC) resin with a proportion of 95% indene, *M*_w_ = 1092 g/mol, PDI = 3.07.

**Table 2:** Amount of resin in phr and wt% as well as the amount of the total rubber compound in phr.

Amount Resin [phr]	Amount Total Mixture [phr]	Amount Resin [wt%]
0	178.3	0
20	198.3	10.1
40	218.3	18.3
60	238.3	25.2
80	258.3	31.0

**Table 3:** Vulcanization times *t*_90_ for the SBR compounds with variating resin content.

Amount Resin [phr]	*t*_90_ [min]	
	AMS	IC
0	13	
20	18	14
40	19	17
60	21	19
80	22	20

**Table 4:** Intensity of the glass transition of the pure resins and the calculated and fitted *k* values.

Resin	$\Delta c_{p,r}[J/\mathrm{gK}]$	*k*_0_	*k*_fit_
AMS	0.35	0.65	0.44
IC	0.33	0.69	0.30

**Table 5:** VFTH parameters of fitting the combined data in Figure 12 with Equation (9). The fragility index *m* is calculated using Equation (10).

Sample	*A*	*B* [K]	*T*_v_ [K]	*m*	*T*_g_ (100 mHz) [°C]
Without resin	10.4	355	186	92	−59
20 AMS	10.8	397	189	91	−52
20 IC	10.7	379	191	94	−52
40 AMS	9.3	242	208	121	−44
40 IC	10.2	316	200	106	−47
60 AMS	9.1	222	214	129	−39
60 IC	9.7	277	206	114	−43
80 AMS	8.9	197	220	142	−35
80 IC	9.6	269	208	115	−41

## Data Availability

Restrictions apply to the availability of these data. Data are available from the corresponding author with the permission of Continental Reifen Deutschland GmbH.
